# SARC‐F as a screening tool to detect computed tomography‐based sarcopenia and myosteatosis among older adults with cancer

**DOI:** 10.1002/cam4.6599

**Published:** 2023-11-02

**Authors:** Daniel L. Hess, Christian Harmon, Smita Bhatia, Grant R. Williams, Smith Giri

**Affiliations:** ^1^ Department of Medicine University of Alabama at Birmingham Birmingham Alabama USA; ^2^ Institute for Cancer Outcomes and Survivorship University of Alabama at Birmingham Birmingham Alabama USA; ^3^ Division of Pediatric Hematology‐Oncology, Department of Pediatrics University of Alabama at Birmingham Birmingham Alabama USA; ^4^ Division of Hematology and Oncology, Department of Medicine University of Alabama at Birmingham Birmingham Alabama USA

**Keywords:** cancer, computed tomography, myosteatosis, SARC‐F, sarcopenia

## Abstract

**Background:**

The European Working Group on Sarcopenia in Older People (EWGSOP) recommends SARC‐F as a tool for identifying sarcopenia among older adults. However, the role of SARC‐F among older adults with cancer remains unexplored. We aimed to evaluate the diagnostic utility of SARC‐F to identify those with sarcopenia, or low muscle mass (using skeletal muscle index [SMI]), and myosteatosis (using skeletal muscle density [SMD]) from computed tomography (CT) imaging and the association of SARC‐F with all‐cause mortality.

**Methods:**

Older adults (≥60 years) presenting for initial consultation at UAB medical oncology clinic who underwent geriatric assessment were enrolled in a prospective cohort study. We identified study participants who completed SARC‐F screening and had available CT imaging within 60 days of study enrollment. Using single‐slice CT images at the L3 vertebral level, we computed SMI and SMD using published methods. Sarcopenia and myosteatosis were defined using published cutpoints. We calculated the sensitivity and specificity of SARC‐F for detecting low muscle mass and low muscle density using published thresholds. Finally, we computed the impact of SARC‐F and CT measures on overall survival using Kaplan–Meier curves and Cox regression models, after adjusting for age, sex, cancer type, and cancer stage.

**Results:**

We identified 212 older adults with a median age of 68.8 years; with 60.8% males, 76.6% whites, and pancreatic cancer (21.2%) being the most common malignancy. In the overall cohort, 30.7% had abnormal SARC‐F using published cutpoints. SARC‐F ≥ 4 had a sensitivity of 35% and a specificity of 76% to identify low muscle mass. SARC‐F ≥ 4 had a sensitivity of 38% and a specificity of 74% to identify low muscle density. Those with SARC‐F ≥ 4 and low SMI/SMD had worse survival compared to those with low SMI/SMD alone. Incorporating SARC‐F improved survival prognostication beyond SMI and SMD (HR = 3.1; *p* < 0.001; Harrel's C from 0.73 to 0.76).

**Conclusions:**

SARC‐F as a screening tool has limited diagnostic utility for identifying older adults with low muscle mass and/or density. However, SARC‐F retains prognostic value independent of CT‐based muscle measures in predicting mortality among older adults with cancer.

## Graphical abstract

Patient‐reported measures such as SARC‐F have been used to identify sarcopenia in community‐dwelling adults, but the utility of SARC‐F to identify sarcopenia and myosteatosis among older adults with cancer remains unknown. Herein, we show that SARC‐F and computed tomography analysis identify distinct populations, and they can be used together to better predict overall survival among older adults with cancer.

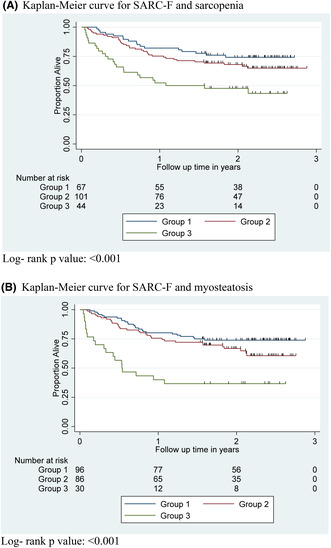

## 1 INTRODUCTION

The majority of cancer and death from cancer occurs in older adults ≥65 years of age.^1^ Among older adults with cancer, there is heterogeneity in treatment tolerability, treatment response, and overall survival. This heterogeneity in outcomes is likely driven by many factors, including differences in frailty status and body composition of older adults. Sarcopenia, or low muscle mass and strength, and myosteatosis, or skeletal muscle fat infiltration, increase longitudinally with aging and are highly prevalent in older adults with cancer.^2^, ^3^, ^4^, ^5^ Sarcopenia is associated with increased chemotherapy‐induced toxicities, increased hospital length of stay, worse treatment tolerability, and increased mortality across multiple cancer types.^6^, ^7^, ^8^, ^9^, ^10^, ^11^, ^12^ Similarly, myosteatosis is associated with functional impairments and decreased overall survival across multiple cancer types.^4^, ^13^, ^14^ Therefore, sarcopenia and myosteatosis are both negatively correlated with functional outcomes and survival in older adults with cancer.

Despite being recognized as key prognostic factors in cancer survival, sarcopenia and myosteatosis remain challenging to quantify and apply in routine cancer care. Whereas sarcopenia and myosteatosis can be quantified through various imaging modalities, computed tomography (CT) is most commonly used among patients with cancers, since most patients routinely get CT imaging as part of their cancer care.^15^, ^16^, ^17^ Herein, we define sarcopenia as CT‐measured low muscle mass and define myosteatosis as CT‐measured low muscle density. Although reliable methods exist for quantifying muscle mass and muscle density using CT imaging, this requires specialized software, trained staff, and is not yet routinely available clinically as a part of radiology workflow. Recognizing the difficulty in identifying sarcopenia in routine clinical practice, SARC‐F, a 5‐item patient‐reported questionnaire, has been recently proposed as a screening tool for sarcopenia.^18^, ^19^, ^20^ SARC‐F stands for Strength, Assistance in Walking, Rise from a chair, Climb Stairs, and Falls. Each of the five components is scored between 0 and 2, for a total score between 0 and 10 (Table S1). A score ≥4 has been shown to have low sensitivity but high specificity for identifying sarcopenia in community‐dwelling adults.^21^, ^22^

However, the utility of SARC‐F to identify sarcopenia in older adults with cancer remains unexplored. To that end, we have previously reported our initial experience in using SARC‐F in oncology patients and showed that SARC‐F ≥ 4 was associated with increased mortality in a population of older adults with cancer.^23^ Here, we sought to assess the diagnostic utility of SARC‐F to identify sarcopenia (low muscle mass on CT imaging) and myosteatosis (low muscle density on CT imaging) specifically among older adults with cancer. Additionally, we sought to determine whether combining information on SARC‐F score and CT‐measured muscle mass or density could identify subpopulations with distinct survival outcomes.

## 2 METHODS

### 2.1 Study population

We used data collected from the University of Alabama at Birmingham (UAB) Cancer and Aging Resilience Evaluation (CARE) Study, an ongoing single institution prospective registry of adults ≥60 years old undergoing cancer care (solid tumors and hematologic malignancies) at UAB hospitals and clinics since September 2017.^24^ All participants in the CARE study undergo a patient‐reported geriatric assessment (CARE GA) at the time of initial consultation with their medical oncologist. Between May 2019 and August 2020, the five‐item SARC‐F questionnaire was included as an amendment to our original CARE GA instrument. As such, all participants who participated in CARE during this time period also underwent SARC‐F assessment. For the purposes of this study, we included study participants with available SARC‐F scores who also had available CT scans obtained as a part of their cancer care, within 60 days of completion of baseline CARE GA. Therefore, only patients who participated in the CARE study and had a SARC‐F with available CT scans were included in this study (Figure S1). This study was approved by the Institutional Review Board of UAB (IRB‐300000092). Written informed consent was obtained from all patients included in this study.

### 2.2 SARC‐F questionnaire

All study participants completed the SARC‐F questionnaire at the time of initial consultation with a medical oncologist. SARC‐F is a patient‐reported questionnaire which consists of five components: Strength, Assistance in walking, Rise from a chair, Climb stairs, and Falls.^19^ Each component is scored between 0 and 2 points to give a total score between 0 and 10 (Table S1). A score ≥4 has previously been shown to be highly specific for sarcopenia and to be predictive of poor outcomes.^19^, ^20^, ^21^, ^25^

### 2.3 Measurement of skeletal muscle mass and density

We obtained archived CT scans of the abdominal region within 60 days of baseline CARE GA from the UAB Picture Archiving and Communication System. These CT scans were obtained as a part of routine clinical care and the decision to obtain scans rested with the treating oncologist. A time span of 60 days was considered sufficiently accurate to represent body composition at the time of CARE GA. Using single‐slice CT images at the level of the third lumbar vertebra, we identified skeletal muscle area (SMA) using a combination of outlining and tissue‐specific Hounsfield Unit (HU) thresholds (−29 through +150 HU). Prior studies have shown that cross‐sectional measurements of skeletal muscle at the level of the mid‐L3 vertebra are well‐correlated to whole body muscle and adipose tissue mass.^26^, ^27^ Image annotation and segmentation was performed using Data Analysis Facilitation Suite previously validated image processing software.^28^ All images were manually reviewed by a single, trained reviewer to confirm accuracy. Computed SMA was then normalized for height to generate skeletal muscle index (SMI) (SMI = SMA in cm^2^/height in m^2^). We then calculated skeletal muscle density (SMD), or radio‐attenuation, by averaging the HU of skeletal muscle of the cross‐sectional image. Since the density of skeletal muscle is inversely related to muscle fat content, myosteatosis is reflected in a lower SMD.^29^, ^30^, ^31^ We used previously published thresholds by Martin et al.^17^ of SMI and SMD to identify patients with and without sarcopenia and myosteatosis, respectively (Table S2).

### 2.4 Combining SARC‐F and CT‐measured muscle mass/density

To determine the utility of SARC‐F to combine with sarcopenia or myosteatosis to better predict overall survival, we created three mutually exclusive groups based on both SARC‐F score and sarcopenia/myosteatosis. Patients who had SARC‐F < 4 and did not have sarcopenia/myosteatosis were included in Group 1. Patients who had SARC‐F < 4 and had sarcopenia/myosteatosis *AND* patients who had SARC‐F ≥ 4 and did not have sarcopenia/myosteatosis were included in Group 2. Patients who had SARC‐F ≥ 4 and also had sarcopenia/myosteatosis were included in Group 3.

### 2.5 Overall survival

We obtained information regarding vital status using a combination of medical records and linkage with Accurint database. Overall survival was defined as time between baseline GA to death or last follow‐up. Vital status was updated through May 9, 2022.

### 2.6 Other covariates

Age and gender were self‐reported as part of the CARE tool. Cancer type and cancer stage were obtained from review of the electronic medical record.

### 2.7 Statistical analyses

We used descriptive statistics to define baseline demographics and clinical characteristics of patients. With regard to diagnostic validity, we computed sensitivity and specificity of SARC‐F ≥ 4 using CT‐based sarcopenia or CT‐based myosteatosis as the reference standard. Although we initially focused on SARC‐F score ≥4, we explored if alternative cutpoints of SARC‐F score could improve diagnostic validity using receiver operator characteristic (ROC) curves and Youden's J Statistic/Index (YI = sensitivity + specificity − 1).^32^ For overall survival analysis, we used Kaplan–Meier curves and log‐rank tests to compare survival curves between groups. We evaluated the association between SARC‐F scores and the risk of all‐cause mortality using Cox proportional hazards regression models, adjusting for age, gender, cancer type, and cancer stage. All statistical tests were two‐sided, with α < 0.05 considered statistically significant. We performed statistical analyses using SAS statistical software version 9.4 (SAS Institute Inc) and STATA version 16.0 (StataCorp LLC).

## 3 RESULTS

### 3.1 Baseline demographics, SARC‐F score, and CT measurements

Between May 2019 and August 2020, 1322 patients enrolled in the CARE registry. 736 of these patients had CT imaging completed within 60 days of completion of CARE GA. 212 of these patients also completed the SARC‐F questionnaire and were thus included in the present study (Figure S1). The median age of the cohort was 68.8 years (interquartile range [IQR] 64–74). The majority of the patients were male (60.8%) and non‐Hispanic White (76.6%). The most common cancers included pancreatic (21.2%) and colorectal (13.2%). The majority of cancers were Stage IV (50.9%) (Table 1).

**TABLE 1:** Baseline demographics of cohort, including age, sex, race, cancer type, and cancer stage.

Variable	Value
*N*	212
Age, median (IQR)	68.80 (63.66, 74.00)
Age, category	
Age, 60–65	67 (31.6%)
Age, 66–70	50 (23.6%)
Age, 71–96	95 (44.8%)
Sex	
Female	83 (39.2%)
Male	129 (60.8%)
Race	
Non‐Hispanic White	160 (76.6%)
Non‐Hispanic Black	46 (22.0%)
Hispanic	3 (1.4%)
Cancer type	
Colorectal	28 (13.2%)
Pancreatic	45 (21.2%)
Hepatobiliary	21 (9.9%)
Gastroesophageal	18 (8.5%)
Other GI	26 (12.3%)
Prostate	18 (8.5%)
Lung	24 (11.3%)
Head and neck	18 (8.5%)
Other	14 (6.6%)
Cancer stage	
Stage I	7 (3.3%)
Stage II	35 (16.5%)
Stage III	59 (27.8%)
Stage IV	108 (50.9%)
Unknown	3 (1.4%)

The median SARC‐F score was 2 (IQR 0–4). 30.7% of patients had a SARC‐F score ≥4. The median SMI was 41.5 (cm^2^/m^2^) (IQR 34–49), and the median SMD was 39.5 (HU) (IQR 32–48). 58.5% of patients met criteria for sarcopenia, and 38.1% of patients met criteria for myosteatosis (Table 2).

**TABLE 2:** Distribution of SMI, SMD, and SARC‐F within the cohort.

Variable	Value
*N*	212
Sarcopenia: No	88 (41.5%)
Sarcopenia: Yes	124 (58.5%)
Myosteatosis: No	130 (61.9%)
Myosteatosis: Yes	80 (38.1%)
SARC‐F score, median	2 (0, 4)
SARC‐F < 4	147 (69.3%)
SARC‐F ≥ 4	65 (30.7%)

### 3.2 Validity of SARC‐F tool for diagnosing sarcopenia and myosteatosis

Using the well‐established cutoff of ≥4, SARC‐F had 35% sensitivity, and 76% specificity for identifying sarcopenia (Table 3A). Meanwhile, SARC‐F ≥ 4 had 38% sensitivity and 74% specificity for identifying myosteatosis (Table 3B). We explored alternative cutpoints of SARC‐F score to detect sarcopenia and myosteatosis. Generally, decreasing cutoffs led to improved sensitivity but decreased specificity to detect sarcopenia (Figure S2A) and myosteatosis (Figure S2B). Conversely, increasing SARC‐F score led to decreased sensitivity but increased specificity to detect sarcopenia (Figure S2A) and myosteatosis (Figure S2B). We confirmed that SARC‐F ≥ 4 was the optimal cutoff using Youden's index. Therefore, we used SARC‐F score ≥4 for the remainder of the study.

**TABLE 3:** Sensitivity and specificity of SARC‐F ≥ 4 to detect low SMI (A) and low SMD (B) measured by CT.

	CT: No	CT: Yes	Total
A. Low SMI			
SARC‐F: No	67	80	147
SARC‐F: Yes	21	44	65
Total	88	124	212
B. Low SMD			
SARC‐F: No	96	50	146
SARC‐F: Yes	34	30	64
Total	130	80	210

*Note*: Sensitivity for low SMI was 35% and specificity for low SMI was 76% (A). Sensitivity for low SMD was 38% and specificity for low SMD was 74% (B).

### 3.3 Association of SARC‐F, sarcopenia, and myosteatosis with overall survival

**FIGURE 1:**
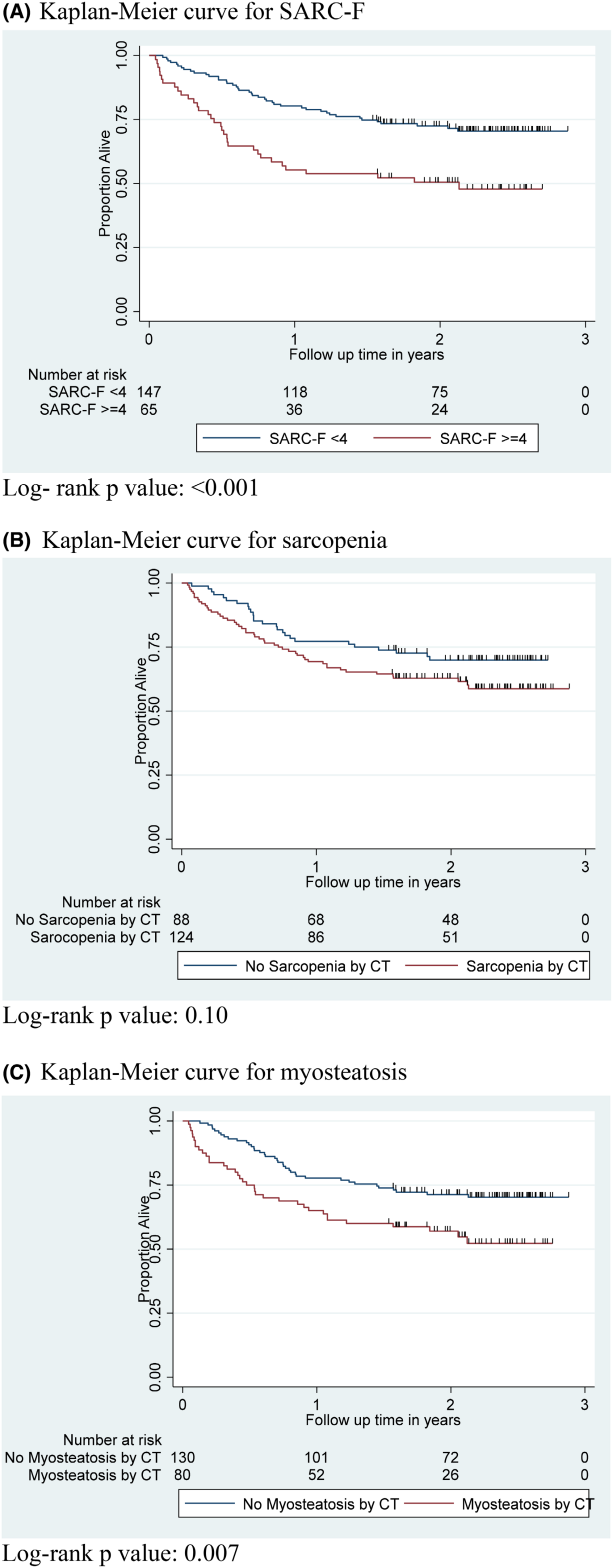
Kaplan–Meier curves demonstrating overall survival of patients based on SARC‐F ≥ 4 (A), low SMI (B), or low SMD (C). (A) Kaplan–Meier curve for SARC‐F. Log‐ rank *p* value: <0.001. (B) Kaplan–Meier curve for sarcopenia. Log‐rank *p* value: 0.10. (C) Kaplan–Meier curve for myosteatosis. Log‐rank *p* value: 0.007.

SARC‐F ≥ 4 was associated with decreased overall survival (*p* < 0.001) (Figure 1A), consistent with our prior report.^23^ We then tested whether sarcopenia and myosteatosis had an impact on mortality in our cohort. Patients with sarcopenia trended toward reduced overall survival, when compared to patients without sarcopenia, but this did not reach statistical significance (*p* = 0.10) (Figure 1B). Patients with myosteatosis had significantly reduced overall survival when compared to patients without myosteatosis (*p* = 0.007; Figure 1C).

**FIGURE 2:**
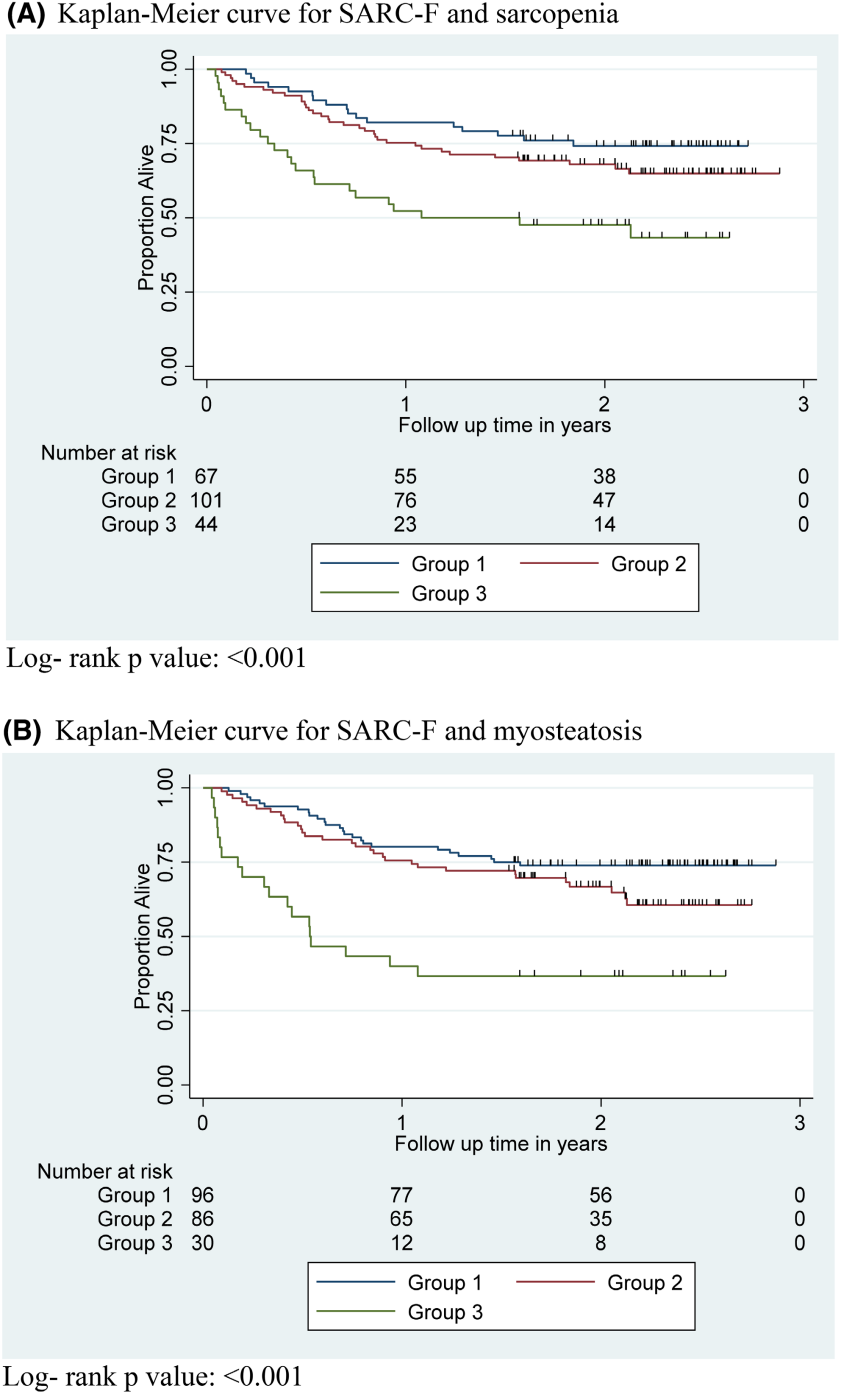
Kaplan–Meier curves demonstrating overall survival of patients based on SARC‐F score and sarcopenia (A) or SARC‐F score and myosteatosis (B). For (A), Group 1 included patients with SARC‐F < 4 who did not have sarcopenia. Group 2 included patients with SARC‐F < 4 who had sarcopenia *and* patients with SARC‐F ≥ 4 who did not have sarcopenia. Group 3 included patients with SARC‐F ≥ 4 who had sarcopenia. For (B), groups were organized in the same manner but were grouped based on myosteatosis, rather than sarcopenia. (A) Kaplan–Meier curve for SARC‐F and sarcopenia. Log‐ rank *p* value: <0.001. (B) Kaplan–Meier curve for SARC‐F and myosteatosis. Log‐rank *p* value: <0.001.

Recognizing that SARC‐F ≥ 4 was predictive of decreased overall survival, we hypothesized that the addition of SARC‐F to CT measures could provide a better stratification of survival. To test this hypothesis, we created three mutually exclusive groups based on both SARC‐F score and sarcopenia. Group 1 included patients with SARC‐F < 4 who did not have sarcopenia. Group 2 included patients with SARC‐F < 4 who had sarcopenia *AND* patients with SARC‐F ≥ 4 who did not have sarcopenia. Group 3 included patients with SARC‐F ≥ 4 who had sarcopenia. Patients in Group 3 had significantly decreased overall survival compared to patients in either Group 1 or Group 2 (*p* < 0.001; Figure 2A). Similarly, we created three mutually exclusive groups based on SARC‐F ≥ 4 and myosteatosis. Again, patients in Group 3 had significantly decreased overall survival compared to patients in either Group 1 or Group 2 (*p* < 0.001; Figure 2B).

In order to study the independent impact of SARC‐F on overall survival, we utilized multivariable Cox proportional hazard regression models. SARC‐F remained independently predictive of overall survival (HR 3.03; 95% CI 1.83–5.02; *p* < 0.001) after adjusting for potential confounders including age, sex, race/ethnicity, cancer type, cancer stage, SMI, and SMD (Table 4). Inclusion of SARC‐F led to improved discrimination of our survival model (Harrel's C statistic improved from 0.73 to 0.76).

**TABLE 4:** Impact of SARC‐F scores on overall survival using univariable and multivariable Cox regression models.

SARC‐F ≥ 4 vs. SARC‐F < 4	HR	95% CI of HR	*p* value
Unadjusted	2.24	1.42–3.54	0.001
Model 1^a^	3.29	2.07–5.52	<0.001
Model 2^b^	3.03	1.83–5.02	<0.001

^a^ Adjusted for age, sex, race/ethnicity, cancer type, and cancer stage.

^b^ Adjusted for age, sex, race/ethnicity, cancer type and cancer stage, as well as SMI and SMD as continuous variables.

Because cancer type can impact overall survival, we then limited analysis to high‐risk cancer types associated with decreased survival including pancreatic, hepatobiliary, and gastroesophageal cancers.^33^, ^34^ SARC‐F ≥ 4 was associated with worse overall survival among these cancer types (HR 4.99; CI 1.68–14.83; *p* = 0.004) (Table S3), after adjusting for age, sex, race/ethnicity, cancer stage, SMI, and SMD. Similarly, because cancer stage can impact overall survival,^33^ we then limited analysis to Stage III and Stage IV cancers. SARC‐F ≥ 4 was associated with worse overall survival among Stage III and Stage IV cancers (HR 4.66; CI 1.92–11.33; *p* = 0.001) (Table S3) after adjusting for age, sex, race/ethnicity, cancer type, SMI, and SMD.

## 4 DISCUSSION

Our study suggests that among older adults with cancer, SARC‐F has limited diagnostic utility for identification of patients with low muscle mass and density. Interestingly, however, SARC‐F provided meaningful prognostic information above and beyond what was captured in CT‐based measurements. In particular, patients with sarcopenia and/or myosteatosis who also had an abnormal SARC‐F score had worse overall survival. To our knowledge, our study is the first to specifically evaluate the diagnostic validity of SARC‐F among older adults with cancer.

Previous studies have examined the diagnostic validity of SARC‐F among community‐dwelling older adults. For instance, Woo et al. evaluated the diagnostic validity of SARC‐F among Chinese men and women age 65 and older. Sarcopenia was measured using various working group consensus definitions, where patients were labeled sarcopenic if below thresholds for low muscle mass, strength, and/or physical performance. The authors showed that SARC‐F had sensitivity ranging from 4% to 10% and specificity ranging from 94% to 99%, depending on the definition of sarcopenia that was used.^21^ Similarly, Ida et al. performed a meta‐analysis examining the validity of SARC‐F to detect sarcopenia among community‐dwelling older adults. Sarcopenia was measured using similar working group consensus definitions. They found that SARC‐F had sensitivity ranging from 14% to 21% and specificity ranging from 90% to 94%.^25^ A subsequent meta‐analysis by Voelker et al. showed similar results.^35^ Taken together, our findings are in overall agreement with existing literature which shows that SARC‐F has poor sensitivity but better specificity for detecting sarcopenia. One possible explanation for the low sensitivity of SARC‐F to detect sarcopenia in our study and prior studies is that SARC‐F may better capture measures of low muscle strength and/or physical performance, as opposed to elements of muscle quantity and/or quality.

The European Working Group on Sarcopenia in Older People (EWGSOP2) most recently defined sarcopenia as low muscle strength combined with low muscle quantity or quality. Low physical performance is used to label sarcopenia as severe.^36^ Our study exclusively focused on low muscle mass (CT‐measured sarcopenia) and low muscle density (CT‐measured myosteatosis). Future studies are needed to investigate correlation of SARC‐F with muscle strength and/or physical performance. Our study shows that the SARC‐F questionnaire itself provides valuable prognostic information and is able to identify those older adults with low muscle mass and/or density at greatest risk of mortality. Although the mechanisms underlying this observation are unclear, we hypothesize that SARC‐F is at least partially capturing the functional components of low muscle strength and/or poor physical performance. In support of this hypothesis, SARC‐F is inversely associated with objective measures in strength and physical performance, including reductions in grip strength and gait speed as well as increases in the Timed Up and Go (TUG) Test and 5 chair stand test.^37^, ^38^, ^39^, ^40^, ^41^

Of note, myosteatosis but not sarcopenia was significantly associated with decreased overall survival in our study. This is consistent with prior studies demonstrating that muscle quality, rather than muscle quantity, may better predict mortality, presumably because of its closer association with physical function. For instance, the Health ABC study demonstrated poor correlation of muscle size, but good correlation of muscle strength, with all‐cause mortality.^42^ In a population of older adults with cancer, SMD has been shown to better correlate with physical function impairments, as compared to SMI.^13^

Our study also has several limitations. Data were collected from a single institution in the southeastern United States with predominantly gastrointestinal malignancies and may not be readily generalizable to other populations. Our assessment of sarcopenia was based on CT measurement of muscle mass alone. There is an emerging consensus that the diagnosis of sarcopenia requires demonstration of impaired muscle function/physical performance as well,^36^ which was not measured in our study. We used pre‐published SMI/SMD thresholds to define patients with low muscle mass or density. In reality, these values are on a continuum, and there is no universal consensus on what constitutes a low SMI/SMD. We had limited information about the type of treatment received which may have biased our results. Our study is also limited by small sample size, with a limiting factor being the number of patients who completed the SARC‐F portion of the CARE GA. In particular, we had fairly limited number of patients across multiple cancer types. While our time to event models included cancer type and cancer stage as potential confounders, inclusion of multiple cancer types with few patients may have led to residual confounding and limited statistical power by increasing the number of parameters in the model. As such, we believe future studies are needed to verify our findings in larger and more homogenous cohorts of patients.

In conclusion, our study adds to the body of literature suggesting that SARC‐F has limited diagnostic validity as a sarcopenia screening tool yet can be a useful tool providing valuable prognostic information among older adults with cancer. Future studies should investigate the association of SARC‐F with performance‐based measures of muscle function to better understand its utility in the evaluation of older adults with cancer.

## AUTHOR CONTRIBUTIONS

**Daniel L. Hess:** Conceptualization (equal); data curation (equal); investigation (equal); methodology (equal); visualization (equal); writing – original draft (lead); writing – review and editing (equal). **Christian Harmon:** Data curation (lead); methodology (equal); resources (equal); software (equal); writing – review and editing (equal). **Smita Bhatia:** Conceptualization (equal); project administration (equal); supervision (equal); writing – review and editing (equal). **Grant R. Williams:** Conceptualization (equal); data curation (equal); funding acquisition (equal); investigation (equal); methodology (equal); project administration (equal); writing – original draft (equal); writing – review and editing (equal). **Smith Giri:** Conceptualization (equal); data curation (equal); formal analysis (lead); funding acquisition (equal); investigation (equal); methodology (equal); project administration (equal); writing – original draft (equal); writing – review and editing (equal).

## FUNDING INFORMATION

Supported in part by the Walter B. Frommeyer Fellowship in Investigative Medicine at the University of Alabama at Birmingham and the National Cancer Institute of the National Institutes of Health (K08CA234225). The content is solely the responsibility of the authors and does not necessarily represent the official views of the National Institutes of Health.

## CONFLICT OF INTEREST STATEMENT

The authors have no current or potential conflicts of interest to disclose.

## ETHICS STATEMENT

All authors comply with the ethical guidelines for authorship and publication in cancer.

## Supporting information


**Data S1.** Supporting Information

## Data Availability

All data will be made available upon request after publication of the article.
