# Supplementary material for: SARC‐F as a screening tool to detect computed tomography‐based sarcopenia and myosteatosis among older adults with cancer
Source: Cancer Med. 2023 Nov 2;12(22):20690–8. doi: 10.1002/cam4.6599 (PMC10709718; doi:10.1002/cam4.6599)
Supplement: Supplementary file 1 — Data S1. Supporting Information [file CAM4-12-20690-s001.docx]

**Table S1** 5-item SARC-F questionnaire. Each item is scored between 0 and 2 to give a total score between 0 and 10

| **Component** | **Question** | **Scoring** |
| --- | --- | --- |
| **Strength** | How much difficulty do you have in lifting and carring 10 pounds? | None=0  Some=1  A lot or unable=2 |
| **Assistance in walking** | How much difficulty do you have walking across a room? | None=0  Some=1  A lot, use aides, or unable=2 |
| **Rise from a chair** | How much difficulty do you have transferring from a chair or bed? | None=0  Some=1  A lot or unable without help=2 |
| **Climb stairs** | How much difficulty do you have climbing a flight of 10 stairs? | None=0  Some=1  A lot or unable=2 |
| **Falls** | How many times have you fallen in the past year? | None=0  1-3 falls=1  4 or more falls=2 |

**Table S2** Thresholds for SMI and SMD based on CT image analysis, utilized based on previously published literature

|  | **BMI** | **Men** | **Women** |
| --- | --- | --- | --- |
| Skeletal muscle index (SMI); in cm^2^/m^2^ | Underweight (<20) | <43 | <41 |
|  | Normal weight (20.0 to 24.9) | <43 | <41 |
|  | Overweight (25.0 to 29.9) | <53 | <41 |
|  | Obese (>30) | <53 | <41 |
| Skeletal muscle density (SMD); in HU | Underweight (<20) | <41 | <41 |
|  | Normal weight (20.0 to 24.9) | <41 | <41 |
|  | Overweight (25.0 to 29.9) | <33 | <33 |
|  | Obese (>30) | <33 | <33 |

**Fig. S1** Workflow demonstrating how patients were selected for inclusion

UAB Cancer and Aging Resilience Evaluation (CARE) Study: adults > 60 years old completed geriatric assessment at the time of initial consultation with their medical oncologist

May 2019-August 2020: 591 patients were approached for participation

399 patients consented for participation in CARE GA

325 patients also had complete SARC-F data

212 patients also had analyzable CT images and were thus included in the study

**Fig. S2** Receiver operating characteristic (ROC) curves showing sensitivity and specificity of various SARC-F score cutoffs to identify low SMI (a) and low SMD (b)

**a.**

| **Cutpoint** | **Sensitivity (%)** | **Specificity (%)** | **Correctly classified (%)** | **LR+** | **LR-** |
| --- | --- | --- | --- | --- | --- |
| > 0 | 100.00 | 0.00 | 58.49 | 1.00 |  |
| > 1 | 75.81 | 44.32 | 62.74 | 1.36 | 0.55 |
| > 2 | 62.10 | 61.36 | 61.79 | 1.61 | 0.62 |
| > 3 | 40.32 | 72.73 | 53.77 | 1.48 | 0.82 |
| > 4 | 35.48 | 76.14 | 52.36 | 1.49 | 0.85 |
| > 5 | 27.42 | 84.09 | 50.94 | 1.72 | 0.86 |
| > 6 | 16.94 | 89.77 | 47.17 | 1.66 | 0.93 |
| > 7 | 10.48 | 94.32 | 45.28 | 1.85 | 0.95 |
| > 8 | 5.65 | 96.59 | 43.40 | 1.66 | 0.98 |
| > 9 | 2.42 | 98.86 | 42.45 | 2.13 | 0.99 |
| > 10 | 0.00 | 98.86 | 41.04 | 0.00 | 1.01 |
| > 10 | 0.00 | 100.00 | 41.54 |  | 1.00 |

**b.**

| **Cutpoint** | **Sensitivity (%)** | **Specificity (%)** | **Correctly classified (%)** | **LR+** | **LR-** |
| --- | --- | --- | --- | --- | --- |
| > 0 | 100.00 | 0.00 | 38.10 | 1.00 |  |
| > 1 | 81.25 | 41.54 | 56.67 | 1.39 | 0.45 |
| > 2 | 63.75 | 55.38 | 58.57 | 1.43 | 0.65 |
| > 3 | 46.25 | 72.31 | 62.38 | 1.67 | 0.74 |
| > 4 | 37.50 | 73.85 | 60.00 | 1.43 | 0.85 |
| > 5 | 32.50 | 83.08 | 63.81 | 1.92 | 0.81 |
| > 6 | 17.50 | 87.69 | 60.95 | 1.42 | 0.94 |
| > 7 | 15.00 | 95.38 | 64.76 | 3.25 | 0.89 |
| > 8 | 7.50 | 96.92 | 62.86 | 2.44 | 0.95 |
| > 9 | 3.75 | 99.23 | 62.86 | 4.88 | 0.97 |
| > 10 | 1.25 | 100.00 | 62.38 |  | 0.99 |
| > 10 | 0.00 | 100.00 | 61.90 |  | 1.00 |

**Table S3** Impact of SARC-F scores on overall survival using univariable and multivariable Cox regression models when limited to high-risk cancer types and stage III/IV cancers

| **SARC-F ≥ 4 vs SARC-F < 4** | **Overall survival** | | | | | |
| --- | --- | --- | --- | --- | --- | --- |
|  | **Unadjusted HR** | **95% CI of HR** | **P value** | **Adjusted HR** | **95% CI of HR** | **P value** |
| Limited to high-risk cancer types (Pancreatic,  Hepatobiliary,  Gastroesophageal) | 3.24 | 1.24-8.45 | 0.016 | 4.99^a^ | 1.68-14.83 | 0.004 |
| Limited to stage III and stage IV cancers | 3.21 | 1.48-6.95 | 0.003 | 4.66^b^ | 1.92-11.33 | 0.001 |

^a^ adjusted for age, sex, race/ethnicity, and cancer stage, as well as SMI and SMD as continuous variables

^b^ adjusted for age, sex, race/ethnicity, and cancer type, as well as SMI and SMD as continuous variables
